# Calpain Cleaves Most Components in the Multiple Aminoacyl-tRNA Synthetase Complex and Affects Their Functions*

**DOI:** 10.1074/jbc.M115.681999

**Published:** 2015-08-31

**Authors:** Hui-Yan Lei, Xiao-Long Zhou, Zhi-Rong Ruan, Wei-Cheng Sun, Gilbert Eriani, En-Duo Wang

**Affiliations:** From the ‡ State Key Laboratory of Molecular Biology, Institute of Biochemistry and Cell Biology, Shanghai Institutes for Biological Sciences, Chinese Academy of Sciences, 320 Yue Yang Road, Shanghai 200031, China,; § University of Chinese Academy of Sciences, Beijing 100039, China,; ¶ The School of Life Science and Technology, ShanghaiTech University, 319 Yue Yang Road, Shanghai 200031, China, and; ‖ Architecture et Réactivité de l'ARN, Université de Strasbourg, UPR9002 CNRS, Institut de Biologie Moléculaire et Cellulaire, 15 rue René Descartes, 67084 Strasbourg Cedex, France

**Keywords:** aminoacyl tRNA synthetase, calpain, protein secretion, proteolysis, stress, aminoacylation, protein fragments

## Abstract

Nine aminoacyl-tRNA synthetases (aaRSs) and three scaffold proteins form a super multiple aminoacyl-tRNA synthetase complex (MSC) in the human cytoplasm. Domains that have been added progressively to MSC components during evolution are linked by unstructured flexible peptides, producing an elongated and multiarmed MSC structure that is easily attacked by proteases *in vivo*. A yeast two-hybrid screen for proteins interacting with LeuRS, a representative MSC member, identified calpain 2, a calcium-activated neutral cysteine protease. Calpain 2 and calpain 1 could partially hydrolyze most MSC components to generate specific fragments that resembled those reported previously. The cleavage sites of calpain in ArgRS, GlnRS, and p43 were precisely mapped. After cleavage, their N-terminal regions were removed. Sixty-three amino acid residues were removed from the N terminus of ArgRS to form ArgRSΔN63; GlnRS formed GlnRSΔN198, and p43 formed p43ΔN106. GlnRSΔN198 had a much weaker affinity for its substrates, tRNA^Gln^ and glutamine. p43ΔN106 was the same as the previously reported p43-derived apoptosis-released factor. The formation of p43ΔN106 by calpain depended on Ca^2+^ and could be specifically inhibited by calpeptin and by RNAi of the regulatory subunit of calpain *in vivo*. These results showed, for the first time, that calpain plays an essential role in dissociating the MSC and might regulate the canonical and non-canonical functions of certain components of the MSC.

## Teaser

**Background:** The human multiple aminoacyl-tRNA synthetase complex (MSC) has an elongated and multiarmed structure, which might be sensitive to proteases such as calpain *in vivo*.

**Results:** Partial calpain hydrolysis of MSC components generated specific fragments.

**Conclusion:** Calpain hydrolysis could dissociate the MSC, thereby affecting its function.

**Significance:** Calpain proteolytically degrades MSC components and might regulate the canonical and non-canonical functions of aaRS.

## Introduction

Aminoacyl-tRNA synthetase (aaRS)^2^ catalyzes the specific esterification of an amino acid with the 3′-terminal hydroxyl group of its cognate tRNA. The reaction can be separated into two steps: (*a*) the ATP-PP_i_ exchange for amino acid activation and (*b*) aminoacylation of tRNA (1). Specificity of aminoacylation catalyzed by aaRSs ensures the accurate translation of the genetic code in the first step of protein synthesis (1). In addition to their canonical aminoacylation function, an increasing number of non-canonical (non-catalytic) functions have been identified for aaRS (2, 3).

In the human cytoplasm, a multiple aminoacyl-tRNA synthetase complex (MSC) has been found that comprises 11 polypeptides and harbors the activities of nine aaRSs, bifunctional glutamylprolyl- (GluPro/EP-), isoleucyl- (Ile), leucyl- (Leu), methionyl- (Met), glutaminyl- (Gln), lysyl- (Lys), arginyl- (Arg), and aspartyl- (Asp) tRNA synthetases, and three non-synthetase components, p18, p38, and p43, which are also named AIMP3, AIMP2, and AIMP1, respectively (4).

Over the years, a number of different functions have been proposed for the MSC (5–7). First, the MSC may serve as a means to stabilize its own components. The stability of each component is variable, depending on the presence of other components in the MSC. The AIMPs are critical for the stability of the whole complex (5). Second, the MSC may be considered as a “reservoir” or “depot” for regulatory proteins with auxiliary functions when released (6). Third, the MSC may help eukaryotic cells to create a channel for aminoacyl-tRNAs (7).

Thus, the MSC, as a source of releasable regulatory proteins, is a vital element of cell homeostasis. To date, few data have been reported about the release of the MSC components by protease digestion *in vivo* (4). Protease digestion may be an efficient way to massively release free components of the MSC, which is much more efficient than amino acid modifications or alternative splicing. Using hydrodynamic methods and small angle x-ray scattering, a structural study of the MSC demonstrated that it has an elongated and multiarmed structure with enough space for accessibility of all its components and a large protein surface area for interaction with additional factors, suggesting that the MSC might be easily attacked by proteases *in vivo* (8).

Early studies observed that several mammalian aaRSs with significant parts deleted by controlled proteolysis were fully active, indicating that the deletions were not essential for catalytic activity but were essential for maintaining the structure of the MSC (7). Later, caspase 7 was shown to cleave human MSC proteins *in vitro* (9). p43 was cleaved with the release of the endothelial monocyte-activating polypeptide II fragment, which has inflammatory cytokine properties under apoptotic conditions. Other components, including GluProRS, LysRS, and AspRS, of human MSC were also digested by caspase 7 *in vitro*; however, only ProRS was released from the MSC (9). By contrast, p43-derived apoptosis-released factor, discovered in apoptotic U937 cells, was proposed as a proteolytic fragment of p43 derived from elastase digestion (10). More generally, proteolysis has been observed within several human aaRSs in the specific peptides linking the new accessory domains to the catalytic conserved cores. Interestingly, these linkers are predicted to be random coils and are generally disordered in crystal structures of aaRSs (4).

Ubiquitously distributed, calpain (also called CANP) is a Ca^2+^-dependent family of neutral proteases (11). The activity of calpain is activated by the intracellular Ca^2+^ and inhibited by the endogenous inhibitor calpastatin (12). Among the 15 mammalian calpain isoforms, calpain 1 and calpain 2 are the best characterized: both are heterodimers with a common small regulatory subunit (CAPNS1; also named as calpain 4) and a different large subunit. In the present work, calpain 1 and calpain 2 were chosen as representatives of the distinct, large catalytic subunits (11). Calpain 2, which is also named “m-calpain” or “mm Ca^2+^-requiring calpain,” is a core member of the calpain enzyme family; however, calpain 1 (also named as μ-calpain) requires a lower concentration of Ca^2+^ for activation (μm Ca^2+^-requiring calpain) (11). Compared with other intracellular proteolytic systems, calpains cleave their substrates according to the tertiary structure of substrates rather than their primary sequences (11). Therefore, accessible interdomain unstructured peptide bonds of aaRSs might be favored targets of calpains. In addition, calpain has been reported to regulate protein synthesis by cleaving components of the translation machinery, such as cytoplasmic polyadenylation element-binding protein 3 and eukaryotic initiation factor 4G1 (eIF4G1) (13, 14).

To identify new factors able to release functional fragments *in vivo* from the MSC, with LeuRS as the bait, we performed a yeast two-hybrid screening on a human cDNA library. We found that calpain 2 interacts with LeuRS. Moreover, calpain cleaved other MSC components, which affected their aminoacylation activity and released their specific fragments with potential new functions. Our results suggested that the calpain family is involved in dissociation of the MSC and might regulate the functions of MSC components and connect their canonical and non-canonical functions.

## Experimental Procedures

### 

#### 

##### Materials

Materials for biochemical experiments were reported previously (15). The Matchmaker Gold yeast two-hybrid system was purchased from Takara (Japan); forskolin, l-glutamine, 3×FLAG peptides, and anti-FLAG M2 affinity gel were from Sigma-Aldrich; and l-[^3^H]Gln was from PerkinElmer Life Sciences. Lipofectamine 2000 reagent, puromycin, and fetal bovine serum (FBS) were obtained from Life Technologies, and the cell culture medium was from Biowest (France). Human erythrocyte calpain 1 and calpeptin (*N*-benzyloxycarbonyl-l-leucylnorleucinal), a cell-permeable specific calpain inhibitor, were purchased from Merck; the protease inhibitor mixture was from Roche Applied Science; and the rProtein A/G-agarose was from Abmart Inc. (China). All cell lines were purchased from the cell resource center of the Shanghai Institutes for Biological Sciences, Chinese Academy of Sciences, Shanghai, China.

Antibodies were obtained from different companies. HRP-labeled anti-mouse and anti-rabbit secondary antibodies were purchased from Sigma-Aldrich; the mouse monoclonal antibody against the peptide representing residues 2–27 at the N terminus of calpain 2 was from Santa Cruz Biotechnology, Inc.; rabbit polyclonal antibodies for calpain 1 and the regulatory small subunit CAPNS1 were from Proteintech Group, Inc. (China); the mouse monoclonal antibody against the His_6_ tag was from BD Biosciences; and the mouse monoclonal antibodies against Myc and FLAG epitope were from Abmart Inc. Rabbit polyclonal antibodies directed against LeuRS, IleRS, EPRS, LysRS, p43, p38, and p18 were prepared by the antibody research center of the Shanghai Institutes for Biological Sciences, Chinese Academy of Sciences. Rabbit polyclonal antibodies for ArgRS were prepared by Willget Biotech (China). Human full-length LeuRS, ArgRS, human p43ΔN106, unique domain of human IleRS, mouse full-length p38, p18, LysRS, and WHEP domains (domains present in TrpRS (W), HisRS (H), and GluProRS (EP)) of mouse EPRS were used as antigens for rabbit polyclonal antibody preparation. Mouse antibodies were shown to cross-react with the corresponding human proteins. The mouse monoclonal antibody against human GlnRS (C-terminal region, residues 620–680) was produced by Abiocode, Inc.

##### Construction of Plasmids for Purification of Proteins, Co-immunoprecipitation Experiments, Protein Overexpression, and Immunofluorescence Analysis

The plasmids for protein purification in *Escherichia coli* (BL21) and those for immunoprecipitation or co-immunoprecipitation experiments, protein overexpression, and immunofluorescence analysis in 293T cells were constructed as shown in Table 1.

**TABLE 1:** **Information concerning the different plasmids constructed in this study** LeuRS-CP1, editing domain of LeuRS; GlnRS-N198, GlnRS fragment containing residues 1–198; GlnRSΔN198, GlnRS fragment containing residues 199–775; p43-N106, p43 fragment containing residues 1–106; p43ΔN106, p43 fragment containing residues 107–312; IP, immunoprecipitation; EGFP, enhanced GFP.

Proteins	Tag	Position	Vector
**For overexpression in *E. coli***			
p18, p38, AspRS, ArgRS, LeuRS, LeuRS-CP1, LysRS	His_6_	N terminus	pET28a
p43	His_6_	C terminus	pET30a
GlnRS	His_6_	C terminus	pET30a
	FLAG	N terminus	
Calpain 2	His_6_	C terminus	pET22b
CAPNS1ΔN26*^a^*			pSBETa

**For IP or co-IP experiments**			
p43, p43-N106, p43ΔN106; GlnRS, GlnRS-N198, GlnRSΔN198	FLAG	C terminus	pCDH-CMV-MCS-EF1-puro
Calpain 2, calpain 1	3 × FLAG	C terminus	pCMV-3T3A
LeuRS	Myc	N terminus	pCDNA3
ArgRS, ArgRSΔN63	FLAG	N terminus	pCDNA3
MetRS	His_6_	C terminus	pCDNA3.1
AspRS	Myc	C terminus	pCDNA3.1
p43*^b^*	FLAG	N terminus	pCDNA3.1
EPRS*^c^*	FLAG	N terminus	p3 × FLAG-CMV

**For overexpression in 293T**			
Calpain 2, calpain 1, CAPNS1	3 × FLAG	C terminus	pCMV-3T3A

**For immunofluorescence analysis in 293T**			
GlnRS, GlnRS-N198, GlnRSΔN198	EGFP	C terminus	pEGFP-N2

*^a^* Full-length calpain 2 with CAPNS1 co-overexpressed in *E. coli* could form an inclusion body; therefore we deleted the N-terminal Gly-rich region (∼26 residues) of CAPNS1 to improve the solubility of calpain 2 and named it CAPNS1ΔN26 (16).

*^b^* The vector construction was used for MSC purification.

*^c^* A gift from Paul L. Fox (The Lerner Research Institute).

For CAPNS1 knockdown, we inserted its shRNA into vector pLKO.1. The shCAPNS1 primer sequences were as follows (the core shRNA sequences for targeting CAPNS1 coding sequence (from 350 to 368 bp) are underlined): forward primer, CCGGCCACAGAACTCATGAACATTTCAAGAGAATGTTCATGAGTTCTGTGGTTTTTG; reverse primer, AATTCAAAAACCACAGAACTCATGAACATTTCAAGAGAATGTTCATGAGTTCTGTGG. Random (shcontrol) primer sequences were as follows: forward primer, CCGGCAACAAGATGAAGAGCACCAACTTCAAGAGAGTTGGTGCTCTTCATCTTGTTGTTTTTG; reverse primer, AATTCAAAAACAACAAGATGAAGAGCACCAACTTCAAGAGAGTTGGTGCTCTTCATCTTGTTG.

##### Protein Expression and Purification

We co-purified calpain 2 (large subunit) with CAPNS1ΔN26 (small subunit) by co-expression of their genes in *E. coli* (BL21) according to the method described by Hata *et al.* (16). The purified calpain 2 heterodimer is termed recombinant calpain 2 here. The components of the MSC were purified by expression of their genes from the recombinant plasmids in *E. coli* (BL21) (17). Purification of the MSC by co-immunoprecipitation was performed in 293T cells transfected with the genes encoding FLAG-ArgRS and FLAG-p43 independently. Transfected cells were continuously incubated for 24 h at 37 °C and collected for lysis. Cell lysate supernatants were incubated with anti-FLAG M2 affinity gel for 2 h at 4 °C. The immunoprecipitated complex was washed with PBS buffer four times and then eluted with buffer containing 20 mm HEPES, pH 7.5, 10 mm potassium acetate, 1.5 mm MgAc_2_, and 500 ng/μl 3×FLAG peptides. Eluted MSCs were used as substrates for recombinant calpain 2 or calpain 1 hydrolytic experiments.

##### Yeast Two-hybrid Screening

The gene encoding the C-terminal domain of LeuRS (residues 708–1176) was cloned into pGBKT7 and transformed into yeast Y2HGold strain. The transformed strains were mated with Y187 strains transformed with a simplified human cDNA library. After perfect mating, they were plated on selective plates for positive clone screening. The yeast two-hybrid screening was performed approximately according to the protocol supplied by Takara (Japan).

##### Hydrolysis of MSC Components by Calpain and N-terminal Protein Sequencing

The proteolysis of MSC components (5–20 μg each) was carried out at 37 °C for 30 min in a reaction mixture containing 20 mm HEPES, pH 7.5, 10 mm potassium acetate, 1.5 mm MgAc_2_, and 8 μg/ml recombinant calpain 2 with 5 mm Ca^2+^ or 24 μg/ml calpain 1 with 0.5 mm Ca^2+^ (14). In some reactions, 5 mm EDTA or 100 μm calpeptin was added to chelate Ca^2+^ or inhibit the hydrolytic activity of calpains. After proteolysis, generated fragments from some components, including ArgRS, GlnRS, and p43, were separated by SDS-PAGE and transferred onto PVDF membrane in CAPS transfer buffer. Significant bands were excised, and their N termini were sequenced by Shanghai Applied Protein Technology Co. Ltd.

##### Preparation of Jurkat S100 Extracts

A previous report showed that Jurkat cells have considerable calpain 2, and their cell lysates were used as a calpain 2 source to perform the proteolysis reaction toward substrates (14). Jurkat cells were grown in RPMI 1640 medium supplemented with 10% FBS. The preparation of Jurkat extracts supernatant (Jurkat S100) and the manipulation of protein cleavage by Jurkat S100 were performed according to the method described by Wang and Huang (14).

##### Extraction of p43ΔN106 from U937 Cells

U937 cell starvation was performed as follows. U937 cells were grown with RPMI 1640 medium plus 10% FBS, and then 2.5 × 10^7^ cells were washed with phosphate-buffered saline (PBS) solution twice and cultured with RPMI 1640 medium plus 0.2% FBS for 48 h. Starved U937 cells and culture medium were collected by centrifugation at 240 × *g* for 2 min at 4 °C. After rinsing twice with PBS, cell pellets were lysed with 2× SDS loading buffer. Protein in the culture medium was precipitated according to the method of Park *et al.* (18). The protein extracts from the culture medium and cytosol were separately subjected to Western blotting.

##### Confocal Immunofluorescence Microscopy

293T cells transfected with the plasmids containing the gene encoding GlnRS or its truncated domains with enhanced green fluorescent protein (GFP) at their C termini were fixed in 4% paraformaldehyde and stained with 4′, 6-diamidino-2-phenylindole (DAPI). After washing, immunofluorescence was analyzed under an Olympus FV1000 microscope.

##### Gene Cloning, Transcription, and ^32^P Labeling of tRNA^Gln^(CUG)

The gene encoding tRNA^Gln^(CUG) was recombined into pTrc99b under the control of a T7 RNA polymerase promoter. *In vitro* transcription of tRNA^Gln^(CUG) by T7 RNA polymerase was performed similarly to that described previously (15). The amino acid accepting activity of tRNA^Gln^(CUG) was 1410 pmol/*A*_260_. 3′-End ^32^P labeling of tRNA^Gln^(CUG) was performed as described previously with minor changes (15). Labeling was performed at 37 °C in a mixture containing 60 mm Tris-HCl, pH 8.0, 12 mm MgCl_2_, 15 μm tRNA^Gln^(CUG), 0.5 mm DTT, 15 μm ATP, 50 μm tetrasodium pyrophosphate, 0.333 μm [α-^32^P]ATP, and 10 μm *E. coli* CCA-adding enzyme for 10 min. 0.7 unit/μl pyrophosphatase was added to the mixture for another 5 min. Phenol/chloroform extraction of [^32^P]tRNA^Gln^(CUG) was conducted as described previously (15).

##### tRNA Aminoacylation Assays

Aminoacylation assays were carried out in reaction mixtures containing 30 mm HEPES, pH 7.5, 25 mm KCl, 15 mm MgCl_2_, 5 mm DTT, 4 mm ATP, 20 μm [^3^H]Gln, 10 μm tRNA^Gln^(CUG), and 0.1 mg/ml BSA at 37 °C. Reactions were initiated by adding 20 nm GlnRS or GlnRSΔN198. At various time intervals, aliquots were quenched and precipitated with 5% trichloroacetic acid as described previously (15).

^32^P-labeled tRNA was used to measure the kinetic parameters of aminoacylation because of the high *K_m_* value of the GlnRSΔN198 for Gln (19). The *K_m_* values of the native and mutant GlnRSs for tRNA were measured at 37 °C in a reaction mixture containing 30 mm HEPES, pH 7.5, 25 mm KCl, 15 mm MgCl_2_, 5 mm DTT, 4 mm ATP, 0.1 mg/ml BSA, 0.1 μm [^32^P]tRNA^Gln^(CUG), and 10 or 50 mm Gln for GlnRS or GlnRSΔN198, respectively. Cold tRNA^Gln^(CUG) concentrations varied between 0.1 and 1.7 μm for GlnRS and between 1 and 16 μm for GlnRSΔN198. The reaction was initiated by adding 5 nm GlnRS or 10 nm GlnRSΔN198. The *K_m_* value of GlnRSΔN198 for Gln was assayed by a similar experiment with 0.1 μm [^32^P]tRNA^Gln^(CUG) and 15 μm cold tRNA^Gln^(CUG), and the concentration of Gln varied between 0.05 and 1.6 mm. At specific time intervals, aliquots were removed for quenching in 0.5 m sodium acetate, pH 5.2, and ethanol-precipitated. Sample treatment and data collection were performed as described previously (15). [^3^H]Gln was used to determine the *K_m_* of GlnRS for Gln because of the low *K_m_* values of GlnRS for tRNA and Gln. The reaction contained 30 mm HEPES, pH 7.5, 25 mm KCl, 15 mm MgCl_2_, 5 mm DTT, 4 mm ATP, 10 μm tRNA^Gln^(CUG), and 0.1 mg/ml BSA at 37 °C. The concentration of [^3^H]Gln varied between 2.5 and 25 μm. The reaction was initiated by adding 5 nm GlnRS. Sample treatment and data collection were performed as described previously (15). All experiments were repeated successfully.

## Results

### 

#### 

##### Calpain 2 Interacts with LeuRS and Cleaves Its C Terminus

**FIGURE 1.**
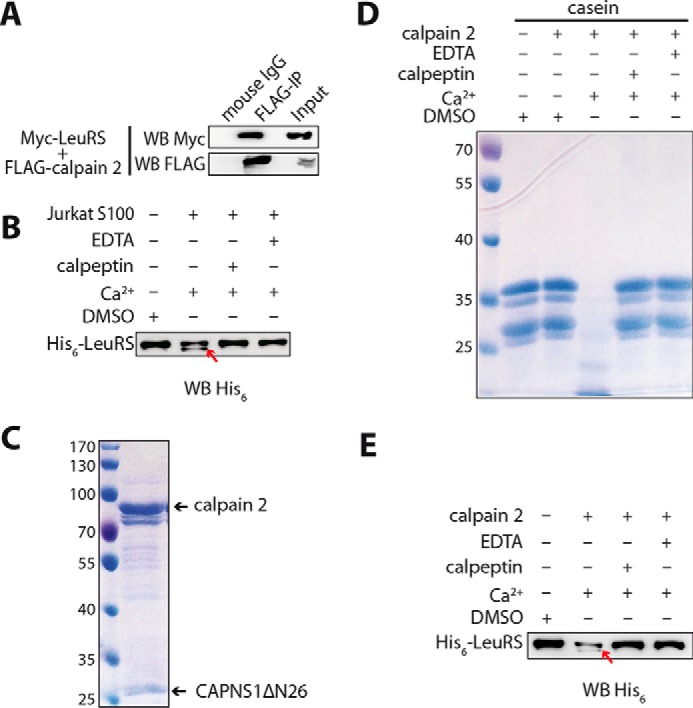
**Partial cleavage of LeuRS by calpain 2.** *A*, LeuRS interacts with calpain 2. The gene encoding FLAG-calpain 2 was co-overexpressed with Myc-LeuRS in 293T cells. Clarified cell lysates were incubated with anti-FLAG antibody for an immunoprecipitation (*IP*) experiment, and co-immunoprecipitated LeuRS was identified by Western blotting (*WB*) with anti-Myc antibody. Normalized mouse IgG was used as a negative control. *B*, LeuRS cleavage by Jurkat lysate supernatant (Jurkat S100) was analyzed. N-terminal His_6_-tagged homogenous LeuRS was used as a substrate, and Jurkat S100 was used as the calpain source. The cleavage is indicated with a *red arrow. C*, purified recombinant calpain 2 was analyzed by Coomassie Blue staining (4.5 μg of protease were loaded). *D*, purified recombinant calpain 2 was activated by calcium and inhibited by the inhibitor calpeptin or EDTA as shown by cleavage of casein. *E*, LeuRS was also partially hydrolyzed by recombinant calpain 2 *in vitro*. The ratio of recombinant calpain 2 to His_6_-LeuRS was 1:50 in the reaction mixture. Cleavage reactions were supplemented with 0.05% (v/v) DMSO, 5 mm Ca^2+^, or 5 mm Ca^2+^ plus 50 μm calpeptin or 5 mm EDTA.

A yeast two-hybrid screen was performed to identify new partners that could interact with the MSC and have potential regulatory functions. Based on our comprehensive biochemical research on LeuRS, human cytosolic LeuRS was chosen as a representative of MSC members, and its C-terminal domain (residues 708–1176) was used as the bait to screen a human cDNA library. 3.4 × 10^6^ clones were screened, and 80 positive clones were isolated. Calpain 2, with 17 occurrences, drew our attention. To verify this interaction, FLAG-calpain 2 was co-expressed with Myc-LeuRS in 293T cells, and a co-immunoprecipitation experiment was performed with anti-FLAG antibody. LeuRS was co-immunoprecipitated with calpain 2 as shown by anti-Myc antibody, confirming the interaction observed in the two-hybrid system (Fig. 1*A*). We used the supernatant of Jurkat extracts (Jurkat S100) containing calpain 2 *in vitro* to assay hydrolysis of recombinant LeuRS (N terminus His_6_-tagged) and found that LeuRS was cleaved under Ca^2+^ stimulation as shown by Western blotting with the His_6_ antibody (Fig. 1*B*). We obtained the purified recombinant calpain 2 containing human calpain 2 (large subunit) and CAPNS1ΔN26 (small regulatory subunit deleted of the first 26 residues) by co-expressing their genes to further confirm the digestion of LeuRS (Fig. 1*C*). The recombinant calpain 2 could cleave casein under Ca^2+^ stimulation (Fig. 1*D*), indicating that it had hydrolysis activity, which could be inhibited by calpeptin or EDTA (calcium chelator) treatment. The recombinant calpain 2 could also hydrolyze the recombinant LeuRS with a pattern similar to that by Jurkat S100 (Fig. 1*E*). Consistently, calpeptin or EDTA completely inhibited the hydrolysis reactions of human LeuRS by recombinant calpain 2 (Fig. 1, *B* and *E*). The data showed that human LeuRS was hydrolyzed by the recombinant calpain 2 indeed.

##### Other Components of Human MSC Are Hydrolyzed by Recombinant Calpain 2

**FIGURE 2.**
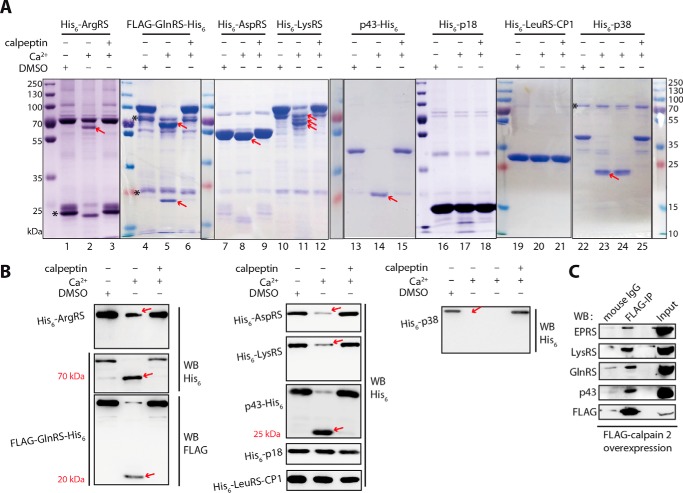
**Cleavages of recombinant MSC components by recombinant calpain 2.** *A*, cleavages of ArgRS, GlnRS, AspRS, LysRS, p43, p18, and p38 by recombinant calpain 2 were analyzed by Coomassie Blue staining. Recombinant proteins were all His_6_-tagged at their N termini, except for p43, which was tagged at its C terminus. For GlnRS, a FLAG or His_6_ tag was located at its N or C terminus, respectively. The ratio of recombinant calpain 2 to recombinant MSC components was 1:50 in the reaction mixture. Mixtures were supplemented with 0.05% (v/v) DMSO, 5 mm Ca^2+^, or 5 mm Ca^2+^ plus 50 μm calpeptin. Proteolytic fragments are indicated by *red arrows* (the cleavage of p38 is shown repeatedly here; *lanes 23* and *24*). Recombinant MSC proteins in addition to recombinant calpain 2 were purified from *E. coli* overexpression systems. After purification, some MSC components remain contaminated with proteins as indicated by *asterisks. B*, the same experiments conducted in *A* were analyzed by Western blotting (*WB*) with antibodies against the N- or C-terminal tags, thereby allowing the identification of the cleavage extremity. The editing domain of human LeuRS (LeuRS-CP1) was used as a negative control for recombinant calpain 2 digestion. Noticeable cleavages are indicated by *red arrows. C*, FLAG-calpain 2 could interact with MSC components *in vivo*. The gene encoding FLAG-calpain 2 was overexpressed in 293T cells, and cell supernatant extracts were incubated with anti-FLAG antibody for immunoprecipitation (*IP*). Co-immunoprecipitated MSC proteins were analyzed by Western blotting with specific antibodies against MSC components (EPRS, LysRS, GlnRS, and p43) or FLAG tag. Normalized mouse IgG was used as a negative control.

We purified seven other components of human MSC by expressing their genes in *E. coli*, except IleRS, MetRS, and EPRS, which had unfavorable expression or solubility (data not shown). In the presence of Ca^2+^ ions or Ca^2+^ ions plus calpeptin inhibitor or EDTA, we analyzed hydrolysis of the seven components by recombinant calpain 2. Except for p18, the other proteins (ArgRS, GlnRS, AspRS, LysRS, p43, and p38) were partially cleaved by the recombinant calpain 2 in a Ca^2+^-dependent manner as detected by Coomassie Blue staining (Fig. 2*A*). The proteolysis reactions were also inhibited by calpeptin (Fig. 2*A*) or EDTA (data not shown). ArgRS (N terminus His_6_-tagged) displayed a truncated peptide very similar in size to its short form generated by translation reinitiation reported in our laboratory (Fig. 2*A*, *lane 2*) (20). Western blotting with anti-His_6_ tag antibody did not reveal any cleaved fragment but did show a reduced amount of full-length ArgRS, suggesting that the cleavage was occurring on its N-terminal extension (Fig. 2*B*). Similarly, recombinant calpain 2 cleaved GlnRS (N/C terminus FLAG-/His_6_-tagged, respectively) into two fragments (∼23 and ∼70 kDa) as demonstrated by Coomassie Blue staining (Fig. 2*A*, *lane 5*). The antibody against His_6_ tag could detect the ∼70-kDa fragment of GlnRS, and the FLAG antibody could detect the ∼23-kDa fragment, showing that the 23-kDa fragment was located in the N-terminal part and the 70-kDa fragment was located in the C-terminal part (Fig. 2*B*). Compared with prokaryotes, human GlnRS, with a molecular mass of about 95 kDa, has a ∼200-amino acid-long N-terminal extension containing the calpain cleavage site, probably located in the unstructured coil linking the N-terminal appended domain. AspRS (N terminus His_6_-tagged) was only cleaved to remove a peptide of about 5 kDa (Fig. 2*A*, *lane 8*); however, Western blotting against His_6_ tag did not reveal any proteolytic fragment, indicating that its N-terminal extension, which is involved in the association with the MSC, was removed (Fig. 2*B*) (21). The recombinant calpain 2 cleaved LysRS (N terminus His_6_-tagged) into three main truncated LysRSs with various N termini deleted (Fig. 2*A*, *lane 11*); Western blotting against the His_6_ tag did not show any fragments (Fig. 2*B*). These data suggested that ArgRS, GlnRS, AspRS, and LysRS were cleaved in their N-terminal extensions and that their intact catalytic cores were retained.

The auxiliary factor p43 (C terminus His_6_-taggged) generated an ∼25-kDa fragment after cleavage by recombinant calpain 2 (Fig. 2*A*, *lane 14*). Western blot analysis with the His_6_ antibody suggested that its N-terminal half was removed (Fig. 2*B*). Similarly, the p38 (N terminus His_6_-taggged) was cleaved, and the generated ∼23-kDa hydrolytic fragment was not detected in a Western blot using the His_6_ antibody (Fig. 2, *A*, *lanes 23* and*24*, and *B*), suggesting that the N-terminal part of p38 was also removed by calpain2. As auxiliary factors of the MSC, p43 and p38 have similar secondary structures formed by a leucine zipper motif in their N-terminal regions and an endothelial monocyte-activating polypeptide II or GST domain in the C-terminal region for p43 or p38, respectively (4). The cleavage pattern showed that their N-terminal regions were all removed by recombinant calpain 2; however, their C-terminal domains of about 25 or 23 kDa were intact (Fig. 2*A*, *lanes 14* and *lanes 23* and *24*). Recombinant calpain 2 did not cleave factor p18 (N terminus His_6_-taggged) as assayed by either Coomassie Blue staining (Fig. 2*A*, *lane 17*) or Western blotting (Fig. 2*B*). As a negative control, the separate editing domain of LeuRS, LeuRS-CP1 (N terminus His_6_-taggged), was stable against hydrolysis by recombinant calpain 2 (Fig. 2, *A*, *lane 20*, and *B*).

To check the *in vivo* relevance of these interactions between calpain 2 and components of the MSC, we overexpressed FLAG-calpain 2 in 293T cells and performed co-immunoprecipitation experiments. We found that EPRS, LysRS, GlnRS, p43 (Fig. 2*C*), and ArgRS and LeuRS (data not shown) could be co-immunoprecipitated with FLAG-calpain 2, indicating that calpain 2 could interact with and cleave MSC components *in vivo*.

##### MSC Components Are Also Hydrolyzed by Calpain 1

**FIGURE 3.**
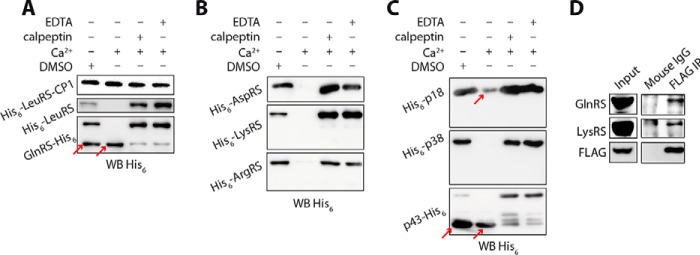
**Analysis of the cleavage of recombinant MSC components by calpain 1.** *A–C*, eight recombinant MSC components were digested by calpain 1, and the hydrolytic products were revealed by Western blotting using an anti-His_6_ antibody. MSC components are basically the same as described in the legend of Fig. 2. The ratio of calpain 1 to MSC proteins was 1:17 in the reaction mixture. The digestion system was supplemented with 0.05% (v/v) DMSO, 0.5 mm Ca^2+^, or 0.5 mm Ca^2+^ plus 50 μm calpeptin or 5 mm EDTA. LeuRS-CP1 was used as negative control for calpain 1 digestion. Noticeable cleavages are indicated by *red arrows. D*, the gene encoding FLAG-calpain 1 was overexpressed in 293T cells, and cell supernatant extracts were incubated with anti-FLAG antibody for immunoprecipitation (*IP*). Co-immunoprecipitated proteins were analyzed by Western blotting (*WB*) with specific antibodies against LysRS and GlnRS. Normalized mouse IgG was used as a negative control.

Calpain 1 and calpain 2 share ∼60% amino acid identity and have similar tertiary structures and recognition specificities toward substrates (11). We used the commercial human erythrocyte calpain 1 to digest MSC components as done for recombinant calpain 2. Calpain 1 could cleave ArgRS, LysRS, AspRS, GlnRS, p43, and p38 in a mode similar to calpain 2 (Fig. 3). Notably, p18 was partially digested, probably in its GST domain, in the N-terminal half (Fig. 3*C*), whereas it was not hydrolyzed by recombinant calpain 2.

The commercial preparation of calpain 1 may contain trace amounts of Ca^2+^ to stimulate the hydrolytic activity; therefore, we observed that it could also hydrolyze these components, except p38 and p18, without extra Ca^2+^ addition. The hydrolytic efficiency without Ca^2+^ addition was in the following estimated order: p43 > LysRS > AspRS > GlnRS > LeuRS/ArgRS > p18/p38 (Fig. 3). More generally, the hydrolysis by calpain 1 was more efficient than that by recombinant calpain 2 and could be further activated by Ca^2+^.

In another assay, calpain 1 expressed in 293T cells was subjected to a co-immunoprecipitation experiment as described in the case of calpain 2. However, EPRS, LeuRS, ArgRS, and p43 failed to be co-immunoprecipitated with FLAG-calpain 1, and only GlnRS and LysRS could be weakly co-immunoprecipitated with calpain 1 (Fig. 3*D*). This result may be the consequence of the transient interaction of calpain 1 with MSC components because this easily activated protease may be rapidly released from these molecules after cleavage. This highly dynamic type of interaction between calpain 1 and its substrates might also explain why calpain 1 was not selected by the yeast two-hybrid screen.

##### Calpains 1 and 2 Can Hydrolyze Most Components in the MSC

**FIGURE 4.**
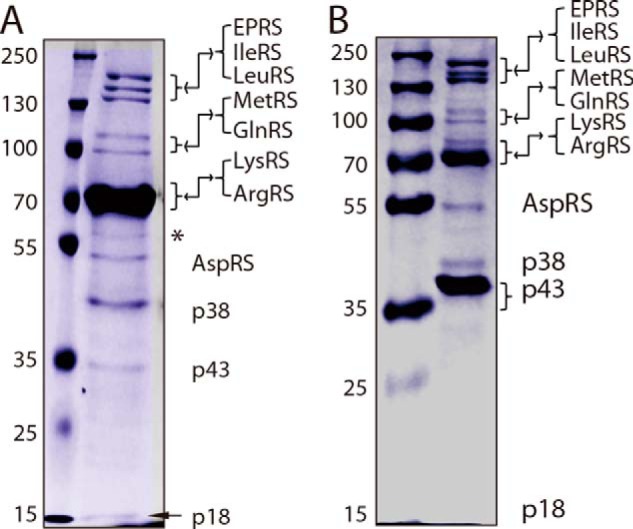
**The MSC purified by co-immunoprecipitation contained the expected 11 components.** *A* and *B*, MSC complexes purified by co-immunoprecipitation were analyzed by Coomassie Blue staining. 293T cells overexpressing ArgRS (*A*) or p43 (*B*) with a FLAG tag at their N termini were harvested. The MSC fraction was purified by co-immunoprecipitation with anti-FLAG affinity gel and eluted with 3×FLAG peptides. An unexpected protein lane is indicated by an *asterisk*. The protein lane of LysRS in *A* was covered by that of ArgRS. p18 in *B* was too faint to be shown clearly by Coomassie Blue staining but could be observed on the Western blots in Fig. 5, *C* and *D*.

**FIGURE 5.**
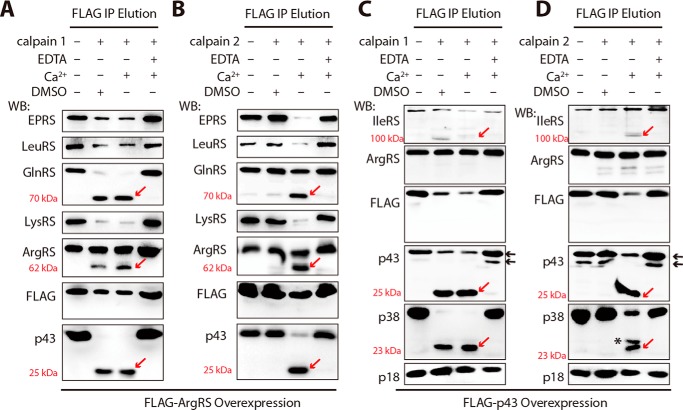
**Components in the MSC are cleaved by calpains 1 and 2.** MSC fractions purified in Fig. 4 were all subjected to cleavage by calpain 1 (*A* and *C*) and recombinant calpain 2 (*B* and *D*). Hydrolysates were analyzed by Western blotting (*WB*) with antibodies specific to each protein. In *C* and *D*, *black arrows* indicate exogenous (*upper*) or endogenous (*lower*) p43. An unexpected fragment, indicated by an *asterisk* (*D*), is probably derived from p38 cleavage by recombinant calpain 2. The concentrations of calpain 1 and recombinant calpain 2 were 3–5 μg/ml; the concentrations of DMSO, Ca^2+^, and EDTA for calpain 1 and 2 were the same, respectively, as described in previous figures. Noticeable cleavages are indicated by *red arrows. IP*, immunoprecipitation.

To determine whether components incorporated into the MSC could be hydrolyzed by calpains 1 and 2, we transfected the gene encoding ArgRS or p43 with a FLAG at its N terminus (FLAG-ArgRS/p43) and overexpressed it in 293T cells independently. Human MSCs were purified by co-immunoprecipitation with anti-FLAG M2 affinity gel. After SDS-PAGE, we observed that the isolated MSCs contained all components as shown by Coomassie Blue staining (Fig. 4). We then subjected the purified MSCs to hydrolysis by calpains. The results of Western blotting with specific antibodies directed against each component showed that in the isolated MSC GluProRS, LeuRS, GlnRS, LysRS, IleRS, p43, and p38, but not p18 and ArgRS, were cleaved by calpain 1 or recombinant calpain 2, which could be deduced from their obviously decreased amounts or generated specific fragments. Cleavage was induced by Ca^2+^ and completely inhibited by EDTA (Fig. 5) or calpeptin inhibitor (data not shown). Most generated fragments of the cleavages of MSC here were similar to those observed from their free forms. Cleavage by calpain 1 or 2 released the catalytic core domain (∼70 kDa) of GlnRS; p43, p38, and IleRS released ∼25-, ∼23-, and ∼100-kDa fragments, respectively (Fig. 5). Notably, the proteolysis results of ArgRS in Fig. 5, *A* and *B*, were different from those in Fig. 5, *C* and *D*. When ArgRS was used as the bait to purify the MSC (Fig. 4*A*), calpains could hydrolyze free ArgRS contaminated in the MSC preparation obtained by immunoprecipitation of the overexpressed ArgRS and generate the short form of ArgRS (Fig. 5, *A* and *B*). However, when p43 was used as the bait for MSC purification (Fig. 4*B*), ArgRS was in the complex and was resistant to proteolysis by calpains (Fig. 5, *C* and *D*). The results indicated that in the MSC ArgRS was resistant to calpain cleavage as was p18. From a recent structural analysis of the ArgRS-GlnRS-p43 complex, this resistance might reflect some steric hindrance of calpain attack that might originate from the N terminus of p43 or the catalytic domain of GlnRS that is in the vicinity of the N terminus of ArgRS in the MSC (22).

**FIGURE 6.**
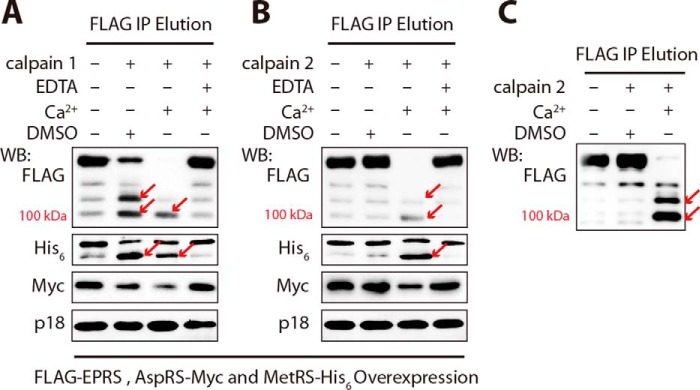
**Other MSC members are also cleaved by calpains 1 and 2.** *A–C*, co-purified MetRS, AspRS, and EPRS were subjected to cleavage by calpain 1 (*A*) or calpain 2 (*B* and *C*). 293T cells co-overexpressing FLAG-EPRS, MetRS-His_6_, and AspRS-Myc were collected for lysis, and the MSCs containing the three proteins were purified by co-immunoprecipitation with anti-FLAG antibody and then subjected to cleavage by calpain 1 (*A*) or calpain 2 (*B* and *C*). p18 was taken as a negative control here. *C*, samples were taken out from *B* earlier for identifying the cleavage extremity of EPRS with anti-FLAG antibody. EPRS was N-terminal FLAG-tagged, and AspRS and MetRS were C-terminal Myc- and His_6_-tagged, respectively. The concentrations of calpain 1, recombinant calpain 2, and supplements were respectively the same as described in Fig. 5. Noticeable cleavages are indicated by *red arrows. WB*, Western blotting; *IP*, immunoprecipitation.

In addition, we co-expressed the genes encoding EPRS with an N-3×FLAG tag, AspRS with a C-Myc tag, and MetRS with a C-His_6_ tag in 293T cells and co-purified AspRS, MetRS, and EPRS with anti-FLAG affinity gel and subjected them to calpain digestion. After digestion by calpains, MetRS produced a large fragment with a deleted peptide of about 20 kDa, probably its N-terminal GST domain (Fig. 6, *A* and *B*); AspRS was truncated in its N-terminal helix (Fig. 6, *A* and *B*); and EPRS produced two major fragments from the N terminus of ∼100 and ∼120 kDa, indicating that the cleavage sites are between the WHEP domains (Fig. 6). The above data showed that, except for ArgRS and p18, calpains 1 and 2 could hydrolyze most components in the MSC.

##### Identification of the Cleavage Sites of Calpains in p43, GlnRS, and ArgRS

**FIGURE 7.**
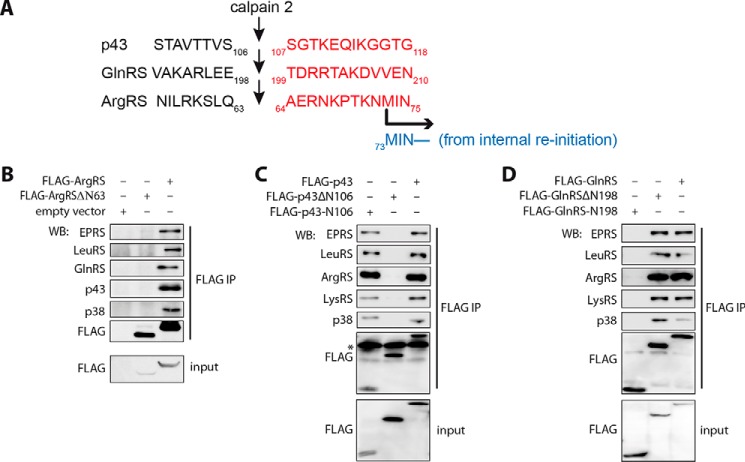
**Identification of cleavage sites of GlnRS, p43, and ArgRS and analysis of their incorporations into the MSC after calpain digestion.** *A*, cleavage sites of GlnRS and p43 by recombinant calpain 2 and of ArgRS by calpain 1 were identified by N-terminal protein sequencing. The sequences in *red* were determined by N-terminal protein sequencing. *B–D*, analysis of the incorporations of truncated domains from GlnRS, p43, and ArgRS into the MSC. Co-immunoprecipitation experiments were performed on 293T cell extracts overexpressing truncated forms of MSC components with FLAG tags. Co-immunoprecipitated proteins were subjected to Western blotting (*WB*) with specific antibodies against MSC components. *ArgRS*Δ*N63*, ArgRS deleted of its first 63 residues; *p43-N106*, p43 from residues 1 to 106; *p43*Δ*N106*, p43 from residues 107 to 312; *GlnRS-N198*, GlnRS from residues 1 to198; *GlnRS*Δ*N198*, GlnRS from residues 199 to 775. An *asterisk* indicates the light chain of the antibody (*C*). *IP*, immunoprecipitation.

Calpains hydrolyze p43, GlnRS in the MSC, and free ArgRS to form certain stable fragments; therefore, we identified their N-terminal sequences, respectively. The N-terminal sequence of the 25-kDa fragment of p43 was ^107^SGTKEQIKGGTG^118^ (Fig. 7*A*), matching perfectly with the sequence of p43 from residues 107 to 118. From the full-length p43 with a molecular mass of 40 kDa, the result showed that calpains cleaved p43 between Ser^106^ and Ser^107^ to form the C-terminal fragment, precisely the same as the previously discovered p43-derived apoptosis-released factor in U937 cells (10). The truncated p43 was named p43ΔN106. Accordingly, that of the 70-kDa truncated GlnRS was ^199^TDRRTAKDVVEN^210^ (Fig. 7*A*), and the cleavage site was between Glu^198^ and Thr^199^. The truncated GlnRS, named GlnRSΔN198, had completely lost its eukaryote-specific N-terminal extension, leaving the intact catalytic core corresponding to bacterial GlnRS (23). Similarly, that of the 62-kDa fragment of ArgRS, with the first amino acid as Ala^64^, showed that the cleavage by calpains was between Gln^63^ and Ala^64^, only nine amino acid residues before the initiation site of ArgRSΔN72 synthesized by translation reinitiation (Fig. 7*A*) (20, 24). The truncated ArgRS was named ArgRSΔN63.

##### Effect of Cleavages by Calpains on Assembly of the MSC

To explore the effect of calpain cleavage on assembly of the MSC *in vivo*, we expressed the genes encoding ArgRS, GlnRS, and p43 or their N/C-terminal domains mimicking the cleavage by calpains in 293T cells and then performed co-immunoprecipitation experiments to analyze the components of MSC. Using a FLAG antibody, two forms of ArgRS were immunoprecipitated, and we observed that EPRS, LeuRS, GlnRS, p38, and p43 could be co-immunoprecipitated with full-length ArgRS but not with ArgRSΔN63 (Fig. 7*B*). These results confirmed that the first 63 residues in the N terminus of ArgRS were critical for its incorporation into the MSC. With p43 and GlnRS, we performed a more comprehensive analysis. We found that p43-N106 (residues 1–106) could interact with the other tested MSC components, including EPRS, LeuRS, ArgRS, LysRS, and p38 (Fig. 7*C*), in agreement with a previous study (25); however, p43ΔN106 could not. GlnRS-N198 (residues 1–198) was only found as a free form in the cell extracts, whereas GlnRSΔN198 was incorporated and could co-immunoprecipitate the other tested components of the MSC (Fig. 7*D*).

##### The Cleavage of GlnRS by Calpains Decreases the Catalytic Efficiency of Its Aminoacylation and Changes Its Localization in Cells

**FIGURE 8.**
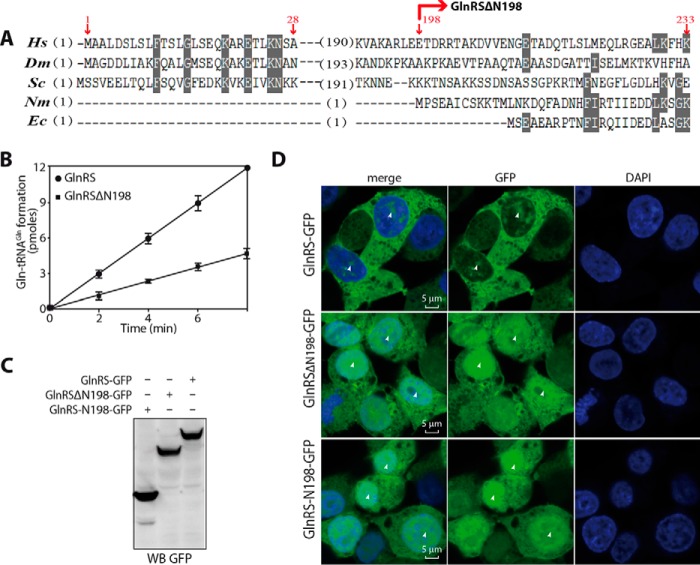
**Comparative analysis of GlnRS with its truncated domains in terms of sequences, aminoacylation activity, and intracellular localization.** *A*, protein sequence alignment of GlnRS from different species. *Hs*, *Homo sapiens*; *Dm*, *Drosophila melanogaster*; *Sc*, *Saccharomyces cerevisiae*; *Nm*, *Neisseria meningitidis*; *Ec*, *E. coli. B*, time course of aminoacylation of tRNA^Gln^ by GlnRSΔN198 and full-length GlnRS. *C*, Western blot showing the expression of GlnRS and its N/C-terminal domains with C-terminal enhanced GFP tags. *D*, immunofluorescence analysis showing the distribution of GlnRS-N198, GlnRSΔN198, and GlnRS in 293T cells after 24 h of expression. Significant differences are indicated with *white arrowheads. WB*, Western blotting. *Error bars* represent S.D.

GlnRS was stable after an unusually large deletion by calpain cleavage, and this prompted us to assay the catalytic efficiency of the truncated GlnRS. By alignment of full-length eukaryotic GlnRS and bacterial GlnRS, human GlnRSΔN198 generated here is comparable in size to the bacterial GlnRS (Fig. 8*A*). Under the same conditions, the aminoacylation activity of full-length GlnRS and GlnRSΔN198 was assayed. The rate of glutaminylation of tRNA^Gln^ catalyzed by GlnRSΔN198 was obviously decreased (Fig. 8*B*). Their kinetic parameters were also measured (Table 2). Compared with those of GlnRS for both substrates, Gln and tRNA^Gln^, the *K_m_* and *k*_cat_ values of GlnRSΔN198 were increased obviously. The data indicated that the affinity of GlnRSΔN198 for the two substrates weakened to 52.3- and 12.87-fold for Gln and tRNA^Gln^, respectively; however, the reaction rate of GlnRSΔN198 increased to 2.5-fold. The data also showed that in the aminoacylation reaction the decreased catalytic efficiency (*k*_cat_/*K_m_*) of GlnRSΔN198 was caused by an elevation of the *K_m_* value.

**TABLE 2:** **Steady-state kinetic parameters for GlnRS and GlnRSΔN198 during aminoacylation** Results are reported as averages of three assays ±S.D.

	GlnRS	GlnRSΔN198
**Gln**		
*k*_cat_ (s^−1^)	0.15 ± 0.05*^a^*	1.12 ± 0.2
*K_m_* (μm)	13.1 ± 1.05*^a^*	685 ± 8
*k*_cat_/*K_m_* (mm^−1^ s^−1^)	11.45*^a^*	1.64

**tRNA^Gln^**		
*k*_cat_ (s^−1^)	0.22 ± 0.05	0.55 ± 0.15
*K_m_* (μm)	0.23 ± 0.21	2.96 ± 0.32
*k*_cat_/*K_m_* (mm^−1^ s^−1^)	956.52	185.81

*^a^* Data were obtained with l-[^3^H]Gln and others with ^32^P-labeled tRNA.

To check whether these deficiencies changed the cellular location of truncated GlnRSs compared with the native form, immunofluorescence analysis was carried out in 293T cells independently expressing GlnRS, GlnRS-N198, and GlnRSΔN198 fused with enhanced GFP (Fig. 8*C*). The full-length GlnRS was mainly located in the cytoplasm with a small amount in the nucleus; however, both GlnRS-N198 and GlnRSΔN198 were accumulated in the nucleus (Fig. 8*D*). The result suggested that the hydrolysis of GlnRS by calpains had a great effect on its cellular distribution, and these generated fragments might be involved in some nuclear processes, such as signaling or transcription, as observed for TyrRS (26).

##### In Vivo Formation of p43ΔN106 Relies on the Proteolytic Activity of Calpain Proteins

**FIGURE 9.**
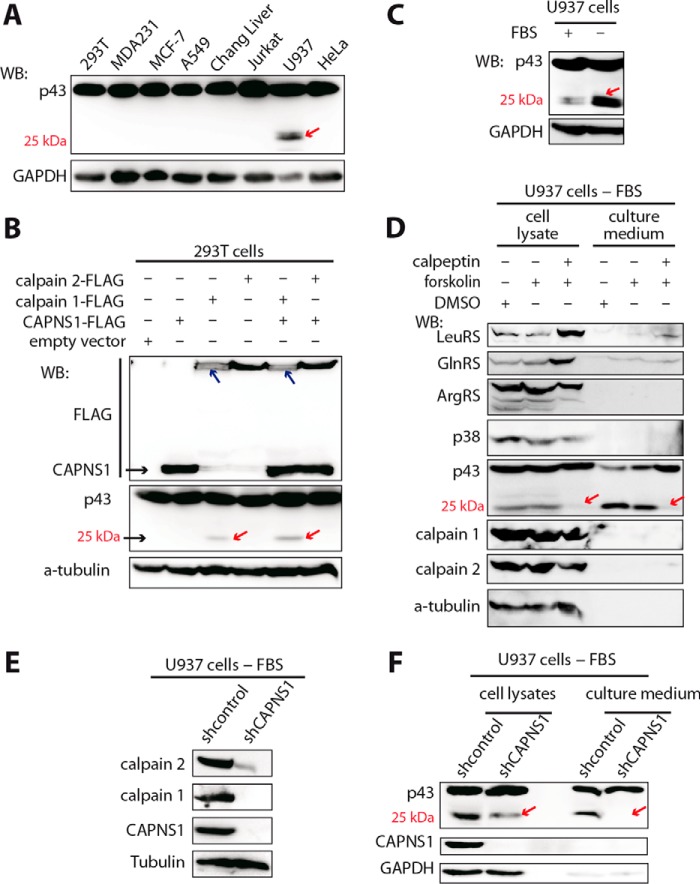
**The generation and secretion of p43ΔN106 *in vivo* are dependent on the hydrolytic activity of calpains.** *A*, p43 cleavage spontaneously occurred in U937 cells. Different cancer cell lines were grown under normal conditions and then harvested for Western blotting (*WB*). Equal amounts of cell lysates were subjected to Western blotting with antibodies directed against p43 and GAPDH. *B*, the cleavage of p43 was detected in 293T cells by overexpression of calpain 1 (*red arrow*). Calpain 1, calpain 2, or CAPNS1 was independently overexpressed in 293T cells for 24 h. Empty vector was used as a negative control. Antibodies for FLAG, p43, and α-tubulin were used for Western blotting. The generation of p43ΔN106 is indicated by *red arrows*. Autolyzed calpain 1 is shown as two bands (*blue arrows*). *C*, serum starvation improved the production of p43ΔN106 in U937 cells. U937 cells were grown under normal or serum starvation conditions and harvested for Western blotting with antibodies directed against p43 and GAPDH. *D*, the production and secretion of p43ΔN106 depended on the hydrolytic activity of calpains. U937 cells were grown under serum starvation conditions and complemented with 0.05% (v/v) DMSO, 50 μm forskolin, or 50 μm forskolin plus 50 μm calpeptin for 48 h. The difference in production and secretion of p43ΔN106, as determined by Western blotting, is indicated with *red arrows*. Antibodies for MSC components including LeuRS, GlnRS, ArgRS, p38, and p43 or antibodies for calpain 1, calpain 2, and α-tubulin were used. The ratio of cell lysates to culture medium loaded for analysis was about 1:4. *E*, Western blot showing the down-regulation of calpain 1 and calpain 2 after CAPNS1 knockdown by RNA interference. Stably transfected U937 cells with an shRNA targeting CAPNS1 or a negative control shRNA were grown under serum starvation conditions for 48 h and harvested for Western blotting. *F*, the production and secretion of p43ΔN106 were impaired by CAPNS1 knockdown. U937 cells with stable CAPNS1 knockdown were serum-starved for 48 h, and then the starved cells and culture medium were harvested and subjected to Western blotting. Differences are shown by *red arrows*.

The proteolytic activity of ubiquitously distributed calpains 1 and 2 is regulated by various factors, such as stress, autolysis, calpastatin, phospholipids, and the Ca^2+^ concentration in the cytoplasm (12). Therefore, under stimulation conditions, calpain cleavages observed *in vitro* may also occur *in vivo* on the components of the MSC. Consequently, we found that among eight different human cell lines p43ΔN106 only existed in U937 cells as assessed by Western blotting against p43 (Fig. 9*A*). To validate that the p43ΔN106 came from calpain cleavage, the gene encoding calpain 1, calpain 2, or regulatory subunit CAPNS1 was overexpressed in 293T cells. P43ΔN106 could be detected when calpain 1, but not calpain 2, was overexpressed probably because the Ca^2+^ concentration in 293T could not reach the mm concentration for activation of calpain 2 (Fig. 9*B*). In addition, partial autolysis in the N terminus of calpain 1 was observed (Fig. 9*B*), which might indicate its activation as described previously (27). This result confirmed that p43ΔN106 could be produced from calpain cleavage *in vivo*.

As the proteolytic activity of calpain is induced by stress conditions (28), we found that without serum in the medium p43ΔN106 in U937 cells was increasingly produced (Fig. 9*C*) and then secreted into the culture medium (Fig. 9*D*). Under serum starvation conditions, we observed that the production (cell lysate) and secretion (culture medium) of p43ΔN106 were completely inhibited by calpeptin. p43 was also reported previously to be secreted with some cytokine function (29); however, its secretion was not inhibited by calpeptin treatment here (Fig. 9*D*). Forskolin, an activator of calpain proteins, acting through cAMP-Ca^2+^-calpain cycle signaling (30), was similarly tested. It did not induce any visible effect on the production or secretion of p43ΔN106, which might be because calpains in starved U937 cells were already fully activated. Besides, under the same conditions, tubulin and other components of the MSC, including ArgRS, LeuRS, GlnRS, and p38, were not detected in the culture medium, indicating that the occurrence of p43ΔN106 in the starvation medium was not from cell lysis but resulted from secretion (Fig. 9*D*). Moreover, calpain 1 or 2 could not be detected in the culture medium, indicating that the cleavage of p43 did not occur after secretion into the starvation medium but occurred earlier in the cytosol (Fig. 9*D*). Previous reports have shown that serum starvation could increase cytosolic Ca^2+^ levels (31, 32), which might activate the hydrolytic activity of calpains, thereby inducing the cleavage of p43.

To further confirm the *in vivo* cleavage, both calpains 1 and 2 were down-regulated by RNA interference of their regulatory subunit CAPNS1 (28). Knockdown of CAPNS1 also induced obvious decreases in calpain 1 and 2 proteins (Fig. 9*E*), which consequently induced a decrease in p43ΔN106 formation in U937 cells and total loss of the secretion into the culture medium (Fig. 9*F*). Unambiguously, this result showed that the p43ΔN106 fragment, which is exactly the same as p43-derived apoptosis-released factor, was formed by calpain cleavage *in vivo* and indicated that calpains could remodel the MSC and release their fragments with new regulatory properties under specific conditions.

## Discussion

**FIGURE 10.**
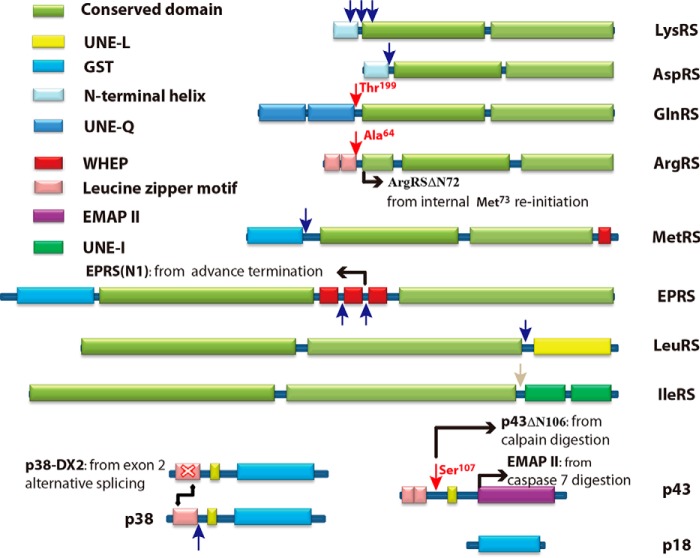
**Proposed locations of calpain cleavage sites of MSC components.** Cleavage sites of GlnRS, ArgRS, and p43 were validated by N-terminal protein sequencing (*red arrows*). Cleavage sites of LeuRS, AspRS, LysRS, GluProRS, MetRS, and p38 were predicted in this study (*blue arrows*). The cleavage site of IleRS was predicted from the three-dimensional structure and the present experimental data (*gray arrows*). *UNE-L/I/Q*, unique domains exclusively found in LeuRS, IleRS, and GlnRS; *ArgRS*Δ*72*, the short form of ArgRS starting from Met^73^ by translation reinitiation from the same mRNA (20); *p38-DX2*, a splicing variant of p38 by alternative splicing leading to exon 2 deletion (50); *EPRS(N1)*, the N-terminal part of EPRS generated from the same mRNA of EPRS by premature translational termination (51); *EMAP II*, endothelial monocyte-activating polypeptide II.

In higher eukaryotic cytoplasm, the elongated and multiarmed structure of the MSC, formed by nine aaRSs and three scaffold proteins, implies high sensitivity to proteolytic cleavage *in vivo* (4, 8). New domains have been progressively appended to aaRSs to improve their own catalytic efficiency, protein-protein association, and even non-canonical functions (5). Initially, through a yeast two-hybrid screen, we observed that calpain 2, a core member of the calpain family, could interact with LeuRS. Further investigations showed that most specific fragments of MSC components could be generated by recombinant calpain 2 and calpain 1. It is thought that the cleavages are mainly localized in the linker peptides of interdomain regions of the components of MSC (Fig. 10). The cleavage could dissociate the MSC and change the catalytic efficiency and cellular localization of some components of the MSC and might connect the canonical and non-canonical functions of aaRS.

### 

#### 

##### Release of MSC Components by Calpain Cleavage

Based on different experimental approaches, the debate concerning MSC components raged for many years until a consensus number of nine aaRSs and three scaffold proteins was decided (4, 7, 33, 34). Interestingly, a longer form of ThrRS (ThrRSL2) was recently reported to be associated with the MSC, potentially increasing the number of aaRS to 10 (35). It has been proposed that *in vivo* the MSC content might be highly dynamic with some components released as free forms (6). However, certain components are essential to maintain the structural integrity of the MSC. For instance, in the N-terminal half of the scaffold protein p38, there is an 83-amino acid sequence that is responsible for the assembly of LysRS, GlnRS, ArgRS, and p43. The remaining part of p38 interacts with the other MSC components (4, 22). Our data showed that p38 cleavage could release a C-terminal fragment of ∼23 kDa, indicating that the potential dissociation of MSC could be induced by calpains *in vivo*. In addition, the three-dimensional structure of the ArgRS-GlnRS-p43 complex showed that the N-terminal domain of GlnRS and C-terminal domain of p43 are very flexible and are probably vulnerable to attack by proteases (22). Indeed, we observed the cleavage of these components by calpains through hydrolytic reaction followed by N-terminal protein sequencing. Cleavages by calpains of the N-terminal parts of MetRS, AspRS, and LysRS; between WHEP domains of EPRS; and of the C terminus of LeuRS also indicated their release from the MSC (4). The N-terminal leucine zipper motif of free ArgRS could also be deleted; however, it could resist digestion by calpains when incorporated into the MSC. Through careful analysis, we hypothesized that the MSCs obtained through purification might exist in a stiff state that is not as relaxed as it is in the cytosol, and the surrounded domains of MSC members, such as GlnRS and p43, could also sterically hinder calpain attack of ArgRS in the MSC. Taken together, these results suggested that the MSC composition could be easily remodeled by calpain cleavage of the flexible peptides linking the accessory domains to the catalytic cores (Fig. 10).

##### Calpain Cleavage Could Affect the Catalytic Parameters of Some Components of the MSC

Since its first discovery four decades ago, many studies have tried to understand the role and benefit of the MSC for eukaryotic cells (5–7). Its primary role is in protein synthesis; therefore, the “channel” theory was first put forward to explain that the MSC formation might help to promote protein synthesis efficiency by conveniently transferring materials from aaRSs to the ribosome (7). The auxiliary protein p43 plays an important role in binding and transferring tRNAs to the aaRSs in the MSC and might also transfer aminoacyl-tRNAs from the MSC to ribosomes (10, 36). However, this assistance might be decreased by calpains under a nutrient-deficient environment.

Our previous investigation on the catalytic characteristics of two forms of ArgRS indicated that they had similar *K_m_* values for their substrates in amino acid activation and aminoacylation reactions; however, the *k*_cat_ values of the short form (ArgRSΔN72) were slightly higher (37). Therefore, compared with ArgRSΔN72, the ArgRSΔN63 generated by calpains with nine redundant residues in the N terminus should have a similar aminoacylation activity. Deletion of the C-terminal extension of LeuRS also had little effect on its catalytic characteristics (37). However, previous comparative kinetic studies for GlnRS in yeast showed that the deleted GlnRS mutant lacking the N-terminal extension exhibited obvious decreased affinity for tRNA^Gln^ and glutamine (23). Here, we also found that the catalytic efficiency of GlnRSΔN198 generated by calpains was decreased in the aminoacylation reaction, caused by the obviously decreased affinity for its substrates. Moreover, the MSC could be easily dissociated by calpains, indicating that the channel formed for aminoacyl-tRNAs transfer was damaged, and consequently, protein synthesis efficiency was impaired.

##### Regulation of Non-canonical Functions of aaRSs and Auxiliary Proteins

Stable macromolecular assemblies, such as the MSC, facilitate complicated, multistep cellular processes. The advantages of these complexes are promoting reaction efficiency and cellular compartmentalization for easier regulation. For many years, the depot hypothesis for the MSC has demonstrated that the MSC could be stimulated to release its components to acquire non-canonical, or “moonlighting,” functions outside the complex (5, 6).

Among the fragments generated by calpains, p43ΔN106 could bind tRNA with higher affinity than native p43 (10), indicating that p43ΔN106 is probably involved in RNA-related regulation. Although p43 is mainly localized in cytoplasm, a recent report showed that p43 could be translocated into the nucleus where it could negatively regulate adipogenesis by inhibiting peroxisome proliferator-activated receptor γ (38). Mirande and co-workers (10) and our laboratory both noted that after the N-terminal deletion p43ΔN106 could easily accumulate in the nucleus. Besides, compared with p43, the cellular localization of GlnRS displays similar characteristics under calpain regulation, indicating that calpain cleavage might affect their possible nuclear regulation functions. Moreover, human full-length GlnRS has a glutamine-dependent antiapoptotic function (39). After cleavage by calpains, the elevated *K_m_* of GlnRSΔN198 for Gln indicated that calpain might be involved in apoptosis through the GlnRS-associated pathway. Currently, there are many similar examples to be considered. Like the three short forms of TrpRS, the two short forms of ArgRS, ArgRSΔN63 (this study) and ArgRSΔN72, could play similar roles in the cell despite their different derivation processes (4).

Starvation could stimulate calpain hydrolytic activity, which is necessary for starvation-induced autophagy (40, 41). Therefore, the inhibition of protein synthesis by calpain cleavage of MSC under starvation may play a critical role in starvation-mediated autophagy. The cleavage of p43 under serum starvation observed here provided some clues in this respect. Contrastingly, calpain was reported to be involved in the mechanistic target of rapamycin-independent autophagy pathway through the cAMP-Ca^2+^-calpain-G_s_α cycle (30). Considering several amino acids (Leu, Gln, Arg, etc.) related to mechanistic target of rapamycin-dependent autophagy signaling (42–45), the proteolysis of MSC components, which may act as principal amino acid sensors, indicates that calpain might also mediate communication between mechanistic target of rapamycin-dependent and -independent autophagy pathways.

##### Calpains Would Be a Good Choice for MSC Regulation

Four major intracellular proteolytic systems exist in organisms, including the ubiquitin-proteasome system, the autophagy-lysosome system, the caspase system, and the calpain family (11). Activated by signal-dependent oligomerization, caspases hydrolyze substrates sequence-specifically mainly for apoptosis (46). Proteasomes and lysosomes constitute the major cellular systems that degrade and eliminate proteins or apparatuses, which could recycle free amino acids for energy and new protein synthesis (47, 48). In contrast, rather than degradation, calpains partially hydrolyze substrates, mainly depending on their three-dimensional structures, in a limited number of sites located between their domains. After proteolysis, substrates would be modulated by calpains for their activity, specificity, localization, longevity, and structures (11). A significant characteristic of this proteolytic process is that the retained parts of substrates could participate in feedback regulation for homeostasis. For example, the Myc-nick fragment resulting from Myc cleavage by calpain can abolish the cell differentiation effect of full-length Myc due to its conserved Myc box region (49). More globally, the hydrolytic regulation by calpains of aminoacyl-tRNA synthesis is reminiscent of that of other factors involved in protein synthesis, such as cytoplasmic polyadenylation element-binding protein 3 and eIF4GI (13, 14). The similar localization of aaRSs and calpain in the cytosol makes fast modulation possible. Furthermore, calpastatin, an endogenous calpain inhibitor, has been co-immunoprecipitated with p43 as revealed by protein mass spectrometry analysis,^3^ which indicated that the hydrolytic activity of calpain on the MSC could be tightly controlled by the close presence of this endogenous calpain inhibitor. Taken together, these observations suggest that under various conditions calpain cleavage offers a way for the cell to dissociate the MSC and to regulate or shut down the protein synthesis machineries, including the components of MSC, and might also release functional fragments that enable the organism to maintain homeostasis.

## Author Contributions

H.-Y. L. and E.-D. W. designed the research. H.-Y. L. performed the research. H.-Y. L., X.-L. Z., Z.-R. R., W.-C. S., G. E., and E.-D. W. contributed to data analysis and proper discussion. H.-Y. L. and E.-D. W. wrote the paper.

## Footnotes

3 H.-Y. Lei and E.-D. Wang, unpublished data.

## Footnotes

2 The abbreviations used are:

aaRS: aminoacyl-tRNA synthetase
MSC: multiple aminoacyl-tRNA synthetase complex
EPRS: glutamylprolyl-tRNA synthetase
WHEP domains: domains present in TrpRS (W), HisRS (H), and GluProRS (EP)
AIMP: aminoacyl-tRNA synthetase complex-interacting multifunctional protein
CAPS: 3-(cyclohexylamino)propanesulfonic acid.
